# Dictyobacter halimunensis sp. nov., a new member of the phylum Chloroflexota, from forest soil in a geothermal area

**DOI:** 10.1099/ijsem.0.006600

**Published:** 2024-12-04

**Authors:** Mazytha Kinanti Rachmania, Fitria Ningsih, Dhian Chitra Ayu Fitria Sari, Yasuteru Sakai, Akira Yokota, Shuhei Yabe, Song-Gun Kim, Wellyzar Sjamsuridzal

**Affiliations:** 1 Department of Biology, Faculty of Mathematics and Natural Sciences, Universitas Indonesia, Kampus UI, Depok 16424, Indonesia; 2 Center of Excellence for Indigenous Biological Resources-Genome Studies, Faculty of Mathematics and Natural Sciences, Universitas Indonesia, Kampus UI, Depok 16424, Indonesia; 3 Department of Microbial Resources, Graduate School of Agricultural Science, Faculty of Agriculture, Tohoku University, 468-1 Aramaki Aza Aoba, Aoba-ku, Sendai, Miyagi 980-8572, Japan; 4 Hazaka Plant Research Center, Kennan Eisei Kogyo Co.Ltd.,, 44 Inariyama, Ashitate, Shibata-gun, Miyagi, 989-1311, Japan; 5 BioResource Research Center, RIKEN, 3-1-1 Koyadai, Tsukuba, Ibaraki, 305-0074, Japan; 6 Biological Resource Center/Korean Collection for Type Cultures (KCTC), Korea Research Institute of Bioscience and Biotechnology, Jeongeup, Jeonbuk 56212, Republic of Korea

**Keywords:** bamboo, *Chloroflexota*, *Dictyobacter halimunensis*, geothermal forest soil, *Ktedonobacteria*

## Abstract

Three Gram-stain-positive aerobic bacteria, characterized by branched mycelia with putative sporangia, were isolated from forest soil inside a decayed bamboo stem from a geothermal area in West Java, Indonesia. The strain S3.2.2.5^T^ grew at 15–37 °C (optimum 30 °C), at pH 5.0–7.0 (optimum 7.0) and in the presence of 0–1% NaCl (optimum 0%). Strain S3.2.2.5^T^ was able to hydrolyse cellulose, xylan, starch and skim milk. The cell-wall sugars were composed of xylose and mannose, and the peptidoglycan hydrolysates contained d-glutamic acid, glycine, d-alanine, l-alanine, *β*-alanine and l-ornithine. The major fatty acids (>10%) were anteiso-C_17:0_, iso-C_17:0_, C_16:1_ 2-OH and iso-C_16:1_. The major polar lipids were phosphatidylinositol, phosphatidylglycerol, diphosphatidylglycerol, unidentified glycolipids and unidentified phospholipids. The major menaquinone was MK-9 (H_2_). The results of the analysis of the phylogenetic tree based on the 16S rRNA gene indicated that these three isolates belong to the genus *Dictyobacter* and they were most closely related to the type strain of species *Dictyobacter aurantiacus* S-27^T^ (97.41–98.00%). The strain S3.2.2.5^T^ exhibited a genome size of 9.41 Mbp, which was significantly larger than the known *Dictyobacter* species. The G+C content was 54.3 mol%. The average nucleotide identity (90.77%) and the digital DNA–DNA hybridization values (42.6%) between strain S3.2.2.5^T^ and *D. aurantiacus* S-27^T^ were below the threshold value for species delineation. Based on the phenotypic, chemotaxonomic and molecular characteristics of strain S3.2.2.5^T^, a novel species of the genus *Dictyobacter*, for which the name *Dictyobacter halimunensis* sp. nov., is proposed. The type strain is S3.2.2.5^T^ (= UICC B-128^T^ = CGMCC 1.61913^T^ = KCTC 43728^T^).

## Introduction

Members of the class *Ktedonobacteria* are recognized as a lineage exhibiting a morphology resembling that of actinomycetes [1], alongside possessing a wealth of gene clusters involved in secondary metabolite biosynthesis [2]. Consequently, these members are regarded as a valuable resource for drug discovery akin to actinomycetes [2]. Members of the class exhibit wide distribution in diverse environments, including common soil [1], volcanoes [3], compost [4], Antarctic soils [5] and cave environments [6]. In our previous study, we demonstrated the abundance of these members in the soil near the Cisolok geysers, a geothermal area in Indonesia [7]. Moreover, we isolated totally 17 *Dictyobacter* strains within the class *Ktedonobacteria* from soil under the bamboo tree and inside a decayed bamboo stem in that area [7,8].

The genus *Dictyobacter*, which belongs to the family *Dictyobacteraceae* in the class *Ktedonobacteria*, was first proposed by Yabe *et al.* [9], with *Dictyobacter aurantiacus* as the type species. Currently, family *Dictyobacteraceae* consists of two genera, which are *Dictyobacter* [9] and *Tengunoibacter* [10]. Until recently, the genus *Dictyobacter* comprises six validly published species (https://lpsn.dsmz.de/genus/dictyobacter), which are *Dictyobacter alpinus* [10], *Dictyobacter arantiisoli* [11], *D. aurantiacus* [9], *Dictyobacter formicarum* [11], *Dictyobacter kobayashii* [10] and *Dictyobacter vulcani* [3].

Members of the genus *Dictyobacter* share common characteristics, including their aerobic, Gram-positive, non-motile, mesophilic natures, and they are characterized by a branched mycelium with spores or sporangiospores [12]. Furthermore, they possess relatively large genomes (7.2–9.2 Mbp) and harbour abundant secondary metabolite biosynthesis gene clusters (11–17 clusters), similar to other members of the order *Ktedonobacterales* [2].

The type species, *D. aurantiacus*, was originally isolated from paddy field soil in Mount Salak, West Java, Indonesia [9]. Other members of the *Dictyobacter* genus have been isolated from alpine regions within volcanoes, a soil-like granular micro-organism mass known as Tengu-no-mugimeshi in Mount Yunomaru and a soil sample collected from Mount Zao in Japan [3,9,11]. Additionally, one species was isolated from an ant house in Honduras [11].

In this study, we conducted characterization of three *Dictyobacter* strains [7,8] and performed experiments to provide a comprehensive taxonomic description of a representative strain. The results of phenotypic, phylogenetic and chemotaxonomic analyses of strain S3.2.2.5^T^ indicated that it represents a novel species of the genus *Dictyobacter*, *Dictyobacter halimunensis* sp. nov.

## Isolation and morphology

Three strains, designated S3.2.2.5^T^, S3.2.1.5 and S3.2.1.6, were previously isolated from soil inside a decayed bamboo stem and under bamboo tree collected in the forest near geysers in Cisolok geothermal area (06°55′991″S, 106°27′187″E), West Java, Indonesia, in November 2018 [8]. Briefly, the Reasoner’s 2A broth (R2A) (Wako Pure Chemical Industries) [13] with 1:10 dilution (pH adjusted to 5.5), supplemented with 60 mg l^−1^ sodium azide (Kanto Chemical Co., Inc.) and solidified by adding 2% (w/v) gellan gum (Kanto Chemical Co., Inc.), was used as an isolation medium. The soil sample was directly streak on the surface of the isolation medium and incubated at 30 °C for 21 days. Purification, at least three times, was conducted on R2A with tenfold dilution and solidified with 2% (w/v) gellan gum. The pure culture was maintained on NITE Biological Resource Center (NBRC) medium 231 with 2% gellan gum at 30 °C. The long-term preservation was carried out by preserving the mycelial suspension at −80 °C in 10% (w/v) skim milk [14] and 20% glycerol solutions [15].

The cultural characteristics of strains S3.2.2.5^T^, S3.2.1.5 and S3.2.1.6 were observed on International Streptomyces Project (ISP) 1, ISP 3 [16], R2A [13], FS1V [17], NBRC medium 231 (0.1% yeast extract, 0.1% beef extract, 0.2% NZ-Amine A, 1.0% maltose and pH 7.0) and each media with tenfold dilution and solidified with 2% (w/v) gellan gum. The morphological properties of colonies of strains S3.2.2.5^T^, S3.2.1.5 and S3.2.1.6 on various media are displayed in Table S1 (available in the online Supplementary Material). Strain S3.2.2.5^T^, S3.2.1.5 and S3.2.1.6 showed abundant substrate mycelia growth only on NBRC 231 solidified with 2% gellan gum. The three strains exhibited a variety of substrate mycelia colours, including pale orange, light yellow orange, yellow orange, orange and bright brown. Moderate growth of these strains was displayed on ISP 1 and tenfold dilution ISP 1, while poor growth was exhibited on ISP 3, tenfold dilution ISP 3, tenfold dilution R2A, FS1V, tenfold dilution FS1V and tenfold dilution NBRC 231, with all media solidified with 2% gellan gum. The strains could not grow on R2A solidified with 2% gellan gum. Notably, the strains could grow on diluted (1/10) R2A but could not grow on full nutrient medium R2A. The soluble pigment was not detected on any media.

**Fig. 1.**
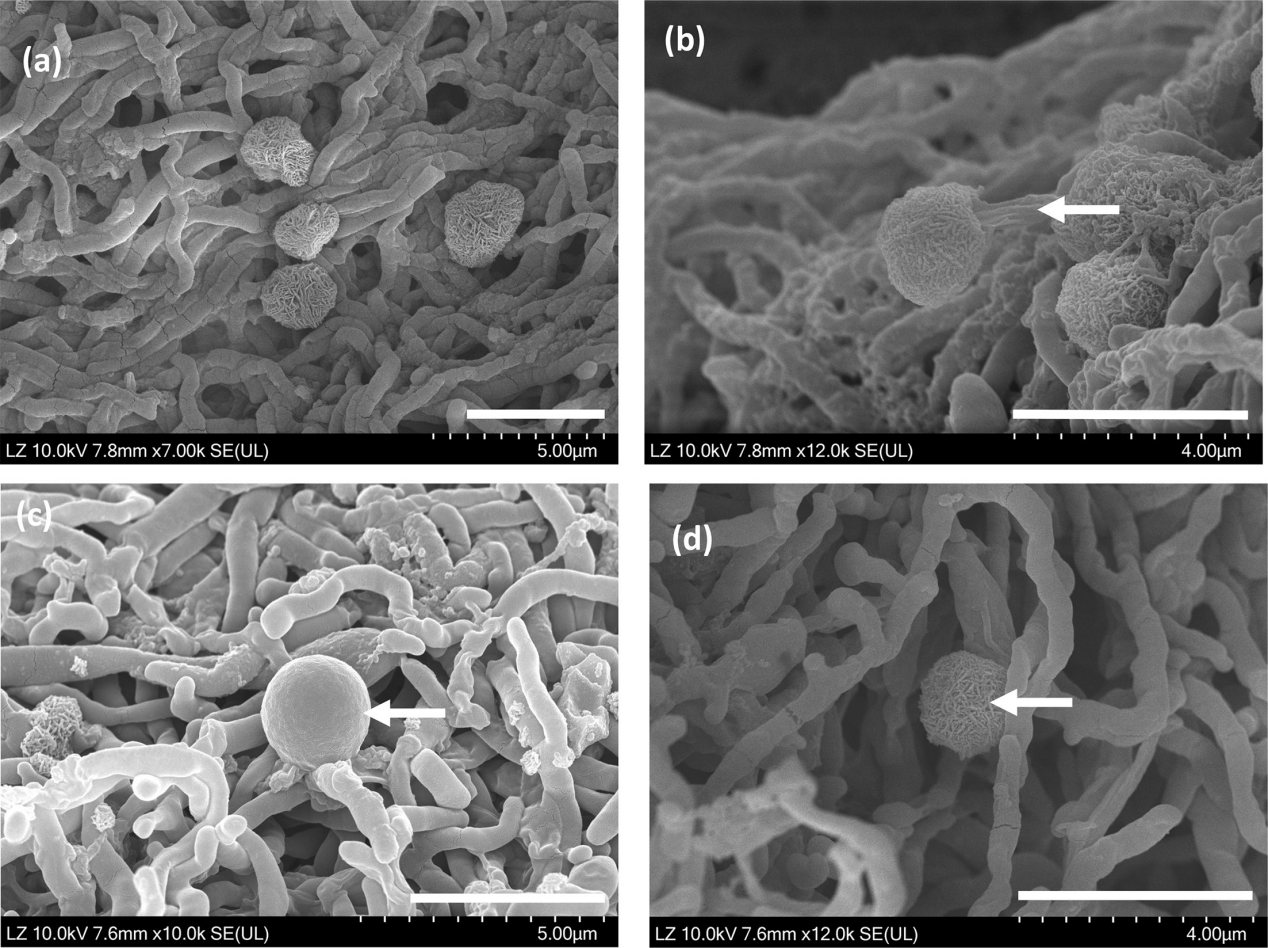
Scanning electron microscope images of strain S3.2.2.5^T^. Putative sporangia structures (**a**), arrow indicated sporangiophore-like structure (**b**), smooth texture of putative sporangium (**c**), mass of fragmented hyphae covered putative sporangium (**d**). All bars, 4 µm.

Scanning electron microscopy (SEM) preparation for strain S3.2.2.5^T^ was conducted according to Le Han *et al.* [18]. Cells were fixed at 4 °C using 2% (w/v) glutaraldehyde in 0.1 M sodium cacodylate buffer, pH 7.4. Cell structures were examined with a Regulus Series FE-SEM, Hitachi. SEM observation showed that strain S3.2.2.5^T^ formed branched mycelia with a 0.5–0.6-µm diameter. Strain S3.2.2.5^T^ forms putative sporangia (Fig. 1a) on NBRC 231 agar after 7 days of incubation at 30 °C under aerobic conditions. These putative sporangia are globose to subglobose, measuring 1.8–3.5 µm in diameter. The sporangiophore-like structures (Fig. 1b), which are 0.5–0.6 µm in diameter, emerge from the vegetative mycelia, grow towards the air and swell at the tip to form putative sporangia. The surface of these structures appears smooth initially (Fig. 1c) and later becomes covered with a mass of fragmented hyphae (Fig. 1d). Sporangia-like structures were also observed in *D. aurantiacus* S-27^T^ [12] and * D. formicarum* SOSP1-9^T^ [11] in their aerial mycelia.

## The 16S rRNA gene and genome-based phylogeny

**Fig. 2.**
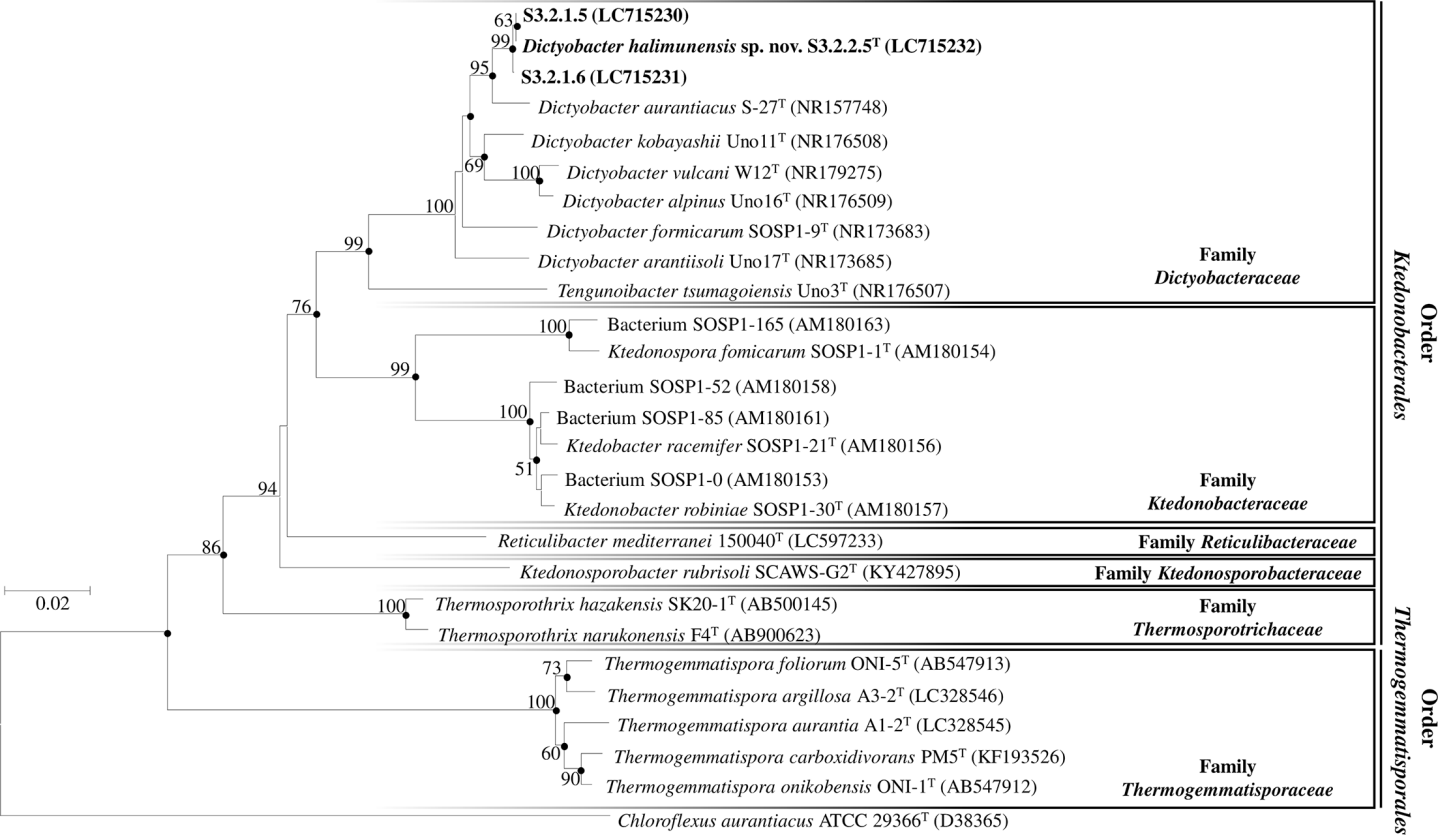
Neighbour-joining tree based on 16S rRNA gene sequence of strain S3.2.2.5^T^, S3.2.1.5 and S3.2.1.6 and all species within the class *Ktedonobacteria.* The tree was constructed based on the 1208 aligned position. *Chloroflexus aurantiacus* ATCC 29366^T^ was represented as an outgroup. Closed circles indicated branches of the tree were found using minimum evolution and maximum likelihood trees. The number at the branched nodes was indicated as a percentage of bootstrap values with 1000 replicates, and only more than 50% of bootstrap values are shown.

Genomic DNA extraction for PCR amplification of the 16S rRNA gene and DNA sequencing of three strains (S3.2.2.5^T^, S3.2.1.5 and S3.2.1.6) was performed in the previous study [2] by using universal *eubacterial* primers of 9F, 536R, 802R, 907F and 1510R. According to the previous studies [7,8], these three strains showed 97.41 (S3.2.1.5 and S3.2.1.6) to 98.00% (S3.2.2.5^T^) of the 16S rRNA gene sequence similarity to *D. aurantiacus* S-27^T^, while to the other strains within the genus *Dictyobacter* were 95.99–96.57% to *D. kobayashii* Uno11^T^, 95.35–96.13% to *Dictyobacter fomicarum* SOSP1-9^T^, 95.51–95.72% to *Dictyobacter arantisolii* Uno17^T^, 95.13–95.49% to *Dictyobacter vulcanii* W12^T^ and 95.13–95.78% to *D. alpinus* Uno16^T^. Among these three strains, the similarity of their 16S rRNA gene sequences was 99.85–99.92%. This indicated that the three strains may belong to the same species (Table S2). The phylogeny tree of the 16S rRNA gene sequences for three strains (Fig. 2) was constructed using mega 11 version 11.0.13 [19] with neighbour-joining [20], minimum evolution [21] and maximum likelihood [22] methods. The reliability of the tree was determined by using 1000 bootstrap replications [23] and the Kimura’s two-parameter method was used to estimate the evolutionary distances [24]. The three isolates were located in a clade adjacent to *D. aurantiacus* with 95% bootstrap value (Fig. 2). The 16S rRNA gene sequence similarity, supported by phylogenetic tree analysis, suggested that these three strains were distinct species from *D. aurantiacus* S-27^T^. Among these three strains, strain S3.2.2.5^T^ was chosen as a representative, and a full taxonomic analysis of this strain was provided in this study.

Strain S3.2.2.5^T^ was cultured in NBRC medium 231 broth for 14 days at 30 °C, 100 r.p.m., and then the cells were harvested by centrifugation. Genomic DNA of strain S3.2.2.5^T^ was extracted from broth culture using the Gentra Puregene Yeast/Bact. Kit (Qiagen) according to the instructions by Zheng *et al.* [2]. The genomic DNA libraries were prepared using kits from Oxford Nanopore Technology (Oxford Science Park, Oxford, UK). Whole-genome sequencing of strain S3.2.2.5^T^ was performed using GridION Flow Cells (R9.4.1) sequencer with the MinKNOW software version 20.06.9 (Oxford Nanopore Technologies). Base calling was carried out with Guppy version 4.0.11 in high-accuracy mode [25]. Filtlong software (https://github.com/rrwick/Filtlong) was used to filter all FASTQ files, and the quality was displayed using NanoPlot [26]. *De novo* assembly was conducted using Flye software version 2.8.1 [27]. The assembled sequence was polished using the Medaka software version 1.2.0 (Oxford Nanopore Technologies Research, https://github.com/nanoporetech/medaka), and then the assembled contig was aligned to the reference genome using Mauve version 2.4.0 [28]. The completeness and contamination levels of the genome sequence were evaluated using the CheckM version 1.1.0 [29].

The annotation of the whole-genome sequence of strain S3.2.2.5^T^ was performed using the DDBJ Fast Annotation and Submission Tool server (https://dfast.ddbj.nig.ac.jp/) [30]. The genome characteristics of strain S3.2.2.5^T^ are shown in Table 1. The complete genome of strain S3.2.2.5^T^ was 9.41 Mbp long and comprised three contigs (3.42 Mbp, 5.91 Mbp and 76.088 bp). The N50 value was 5 914 761 bp. The DNA G+C content was 54.3 mol%. A total of 8030 protein-coding genes were predicted. The numbers of predicted rRNA, tRNA and CRISPRs in the strain S3.2.2.5^T^ genome were 27, 65 and 3, respectively. The completeness value of the genome sequence was 98.28%, and the level of contamination was 6.58% (Table S3).

**Table 1.** The draft whole-genome characteristics, digital DNA–DNA hybridization (dDDH) and average nucleotide identity (ANI) values of S3.2.2.5^T^ and strains of members of the genus *Dictyobacter*. The genome length, G+C content, and number of proteins were obtained from TYGS [46]. The dDDH values (formula *d_4_*) were estimated using the GGDC with the 70% dDDH threshold [33], and the orthoANI value was calculated using the Orthologous ANI Tool software [31] Taxa: 1, *D. halimunensis* sp. nov. S3.2.2.5^T^; 2, *D. aurantiacus* S-27T (GCF_003967515); 3, *D. formicarum* SOSP1-9T (GCF_016587435); 4, *D. kobayashii* Uno11^T^ (GCF_003967555); 5, *D. vulcani* W12^T^ (GCF_ 008974265.1); 6, *D. alpinus* Uno16^T^ (GCF_003967575); 7, *D. arantiisoli* Uno17^T^ (GCF_008326305).

Characteristics	1	2	3	4	5	6	7
Genome length (Mbp)	9.41	8.88	9.21	8.85	7.42	8.96	7.21
DNA G+C content (mol%)	54.3	54.0	51.1	50.3	49.7	49.7	49.6
Number of proteins	8030	7470	7958	8316	6448	7719	5628
dDDH (%) vs. S3.2.2.5^T^	–	42.6	26.2	22.3	21.0	20.7	19.9
ANI (%) vs. S3.2.2.5^T^	–	90.77	81.88	77.80	74.17	73.91	72.04

The orthologous average nucleotide identity (orthoANI) between strain S3.2.2.5^T^ and all species within the genus *Dictyobacter* was calculated using the Orthologous ANI Tool software (https://www.ezbiocloud.net/tools/orthoani) [31]. The orthoANI values of the genomes of all species within the genus *Dictyobacter* were compared to strain S3.2.2.5^T^ (Table 1). The orthoANI values for strain S3.2.2.5^T^ and closely related taxa in the family *Dictyobacteraceae* were 72.04–90.77%, which was lower than the threshold level for species delineation (≥ 95%) [32]. Digital DNA–DNA hybridization (dDDH) was determined by the Type (Strain) Genome Server (TYGS) server based on the Genome-to-Genome Distance Calculator (GGDC) version 3.0 (http://ggdc.dsmz.de) [33,34]. The pairwise dDDH values for strain S3.2.2.5^T^ and closely related taxa in the family *Dictyobacteraceae* were 19.9–42.6% (Table 1), which was lower than the threshold level for species delineation (≥70%) [33].

**Fig. 3.**
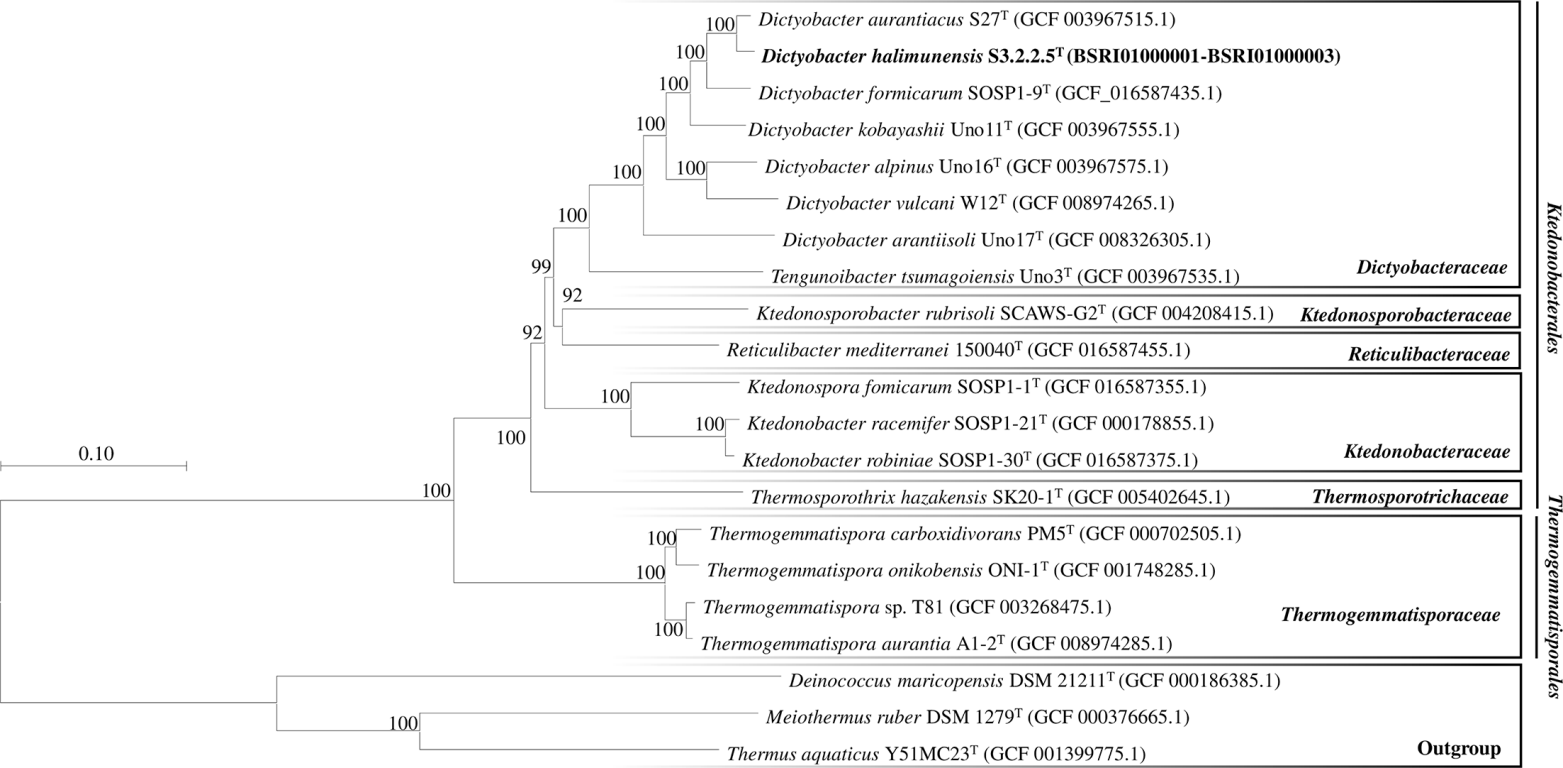
Phylogenomic tree of *D. halimunensis* S3.2.2.5^T^ with the closely related species within the class *Ktedonobacteria* based on the alignment of the bac120 genes. The tree was constructed with the maximum likelihood method using IQ-TREE 2.0.3.

A dataset of 21 genomes consisting of genome sequences of strain S3.2.2.5^T^, type strains of *Ktedonobacteria* species and type strains of *Deinoccocota* species of the phylum *Deinococcota* as outgroup were retrieved from the National Center for Biotechnology Information (NCBI) server (https://www.ncbi.nlm.nih.gov/datasets/genome/). The GTDB-Tk version 2.2.3 [35] was used to identify, extract and align the concatenation of 120 bacterial single-copy marker genes (bac120) from all the genomes. The sequences were trimmed using TrimAI version 1.4.rev15. The sequences were subsequently used to construct a maximum likelihood phylogenetic tree using IQ-TREE 2.0.3 [36] with the best-fit evolutionary rate model: LG+F+R5. The tree was visualized using mega 11 version 11.0.13 [19]. The phylogenomic tree of strain S3.2.2.5^T^ with the closely related species within the class *Ktedonobacteria* is shown in Fig. 3. Strain S3.2.2.5^T^ formed a monophyletic clade with the type strain *D. aurantiacus* S-27^T^, supported by 100% bootstrap value. Genetic data based on the 16S rRNA gene and genome sequences supported that strain S3.2.2.5^T^ represents a new species in the *Dictyobacter* genera within the *Dictyobacteraceae* family.

The antiSMASH version 6.1.1 (https://antismash.secondarymetabolites.org) [37] tool was used to investigate the secondary metabolic gene clusters within the genome of strain S3.2.2.5^T^. Nonribosomal peptide synthase, polyketide synthase and ribosomally synthesized and post-translationally modified peptide family clusters are represented by 13 biosynthetic gene clusters in the genome of strain S3.2.2.5^T^ (Table S4). Ten BGCs from strain S3.2.2.5^T^ were not showing any similarities to the known BGCs, while 3 out of 13 BGCs showed low similarity values (22–28%).

The dbCAN2 meta server (https://bio.tools/dbcan2) [38] tool was used to investigate the carbohydrate-active enzyme within the genome of strain S3.2.2.5^T^ and *D. aurantiacus* S-27^T^ (Table S5). Genome profiling of strain S3.2.2.5^T^ revealed that this strain harbours 307 carbohydrate-active enzymes (CAZymes) in their 9.14 Mbp genome (accounting for 3.82% of the total encoded proteins). A total of 138, 19, 36, 15, 96 and 3 of glycoside hydrolases (GHs), carbohydrate-binding modules (CBMs), carbohydrate esterases (CEs), auxiliary activities (AAs), glycosyl-transferases (GTs) and polysaccharide lyases were predicted in the genome of S3.2.2.5^T^, respectively. *D. aurantiacus* S-27^T^ encoded 266 CAZymes, with a total of 110, 22, 28, 16 and 90 of GHs, CBMs, CEs, AAs and GTs, were predicted in their genome, respectively. Nineteen and 21 CAZymes encoding putative extracellular enzymes were predicted in the genome of strain S3.2.2.5 and *D. aurantiacus* S-27^T^, respectively. The strain S3.2.2.5^T^ currently harbours the largest number of CAZymes in the *Ktedonobacteria* lineage.

## Physiological and biochemical analyses

Growth at various temperatures (15, 23, 30, 37 and 45 °C) was evaluated on NBRC medium 231 gellan gum for 21 days. The salt tolerance to sodium chloride (NaCl) was assessed at 0–4%, with intervals of 0.5% (w/v) NaCl on NBRC 231 agar medium for 21 days incubation at 30 °C. The ability to grow at various pH was observed from pH 3.0 to 8.0 at one pH unit intervals on NBRC 231 broth for 21 days incubation at 30 °C. The pH was adjusted using the following buffering system: 0.1 M acetic acid/0.1 M sodium acetate pH 3.0–4.0, 30 mM MES pH 5.0–6.0 and 30 mM Tris pH 7.0–8.0. The degradation of 0.5% (w/v) carboxymethyl cellulose, microcrystalline cellulose, xylan, starch and skim milk was observed on R2A agar with tenfold dilution for 21 days at 30 °C. Gelatin liquefaction was detected in R2A broth with tenfold dilution supplemented with 10% (w/v) gelatin. The catalase activity was carried out by using 3% hydrogen peroxide (H_2_O_2_) solution. Growth under anaerobic conditions was evaluated by cultivating the strain on NBRC medium 231 gellan gum in an anaerobic chamber (AnaeroPack-Anaero, Mitsubishi Gas Chemical Co., Inc.).

The comparison of physiological and biochemical analyses between the strain S3.2.2.5^T^ and all species within the genus *Dictytobacter* is summarized in Table 2. According to phenotypic investigations, strain S3.2.2.5^T^ was aerobic, Gram reaction-positive and grew at 15–37 °C (optimum at 30 °C), pH 5.0–7.0 (optimum at 7) and 0–1% NaCl in the medium (optimum at 0% NaCl). Carboxymethyl cellulose (CMC), microcrystalline cellulose (MCC), xylan, starch and skim milk were hydrolysed.

**Table 2.** Differential characteristics of strain S3.2.2.5^T^ and related species of the genus *Dictyobacter* Taxa: 1, *Dictyobacter halimunensis* sp. nov. (S3.2.2.5^T^); 2, *D. aurantiacus* S-27^T^ [9]; 3, *D. fomicarum* SOSP1-9^T^ [11]; 4, *D. kobayashii* Uno11^T^; 5, *D. alpinus* Uno16^T^ [10]; 6, *D. arantisolii* Uno17^T^ [11]; 7, *D. vulcani* W12^T^ [3]. All strains are observed on tenfold diluted R2A gellan gum plates for determination of colony colour. Major fatty acids (>10%) and major menaquinone data of strain *D. aurantiacus* S-27^T^ are obtained in this study.

	1	2	3	4		5	6	7
Colony colour	Pale orange	Orange	Pale yellow	Orange		Light grey	Light grey	Pale orange
Sporangium- like structure	+	+	+	Unclear		Unclear	Unclear	Unclear
**Physiological properties**								
Temperature range (°C)	15–37	20–37	20–37	20–37		11–37	20–37	20–37
pH range	5.0–7.0	3.5–8.6	5.0–7.0	4.0–8.0		4.0–8.0	4.0–7.0	5.0–7.0
NaCl tolerance (%, w/v)	1	1	2	1		1	1	1
**Degradative test**								
CMC	+	+	+	+		+	+	−
MCC	+	+	+	+		−	−	w
Xylan	+	+	+	+		−	−	w
Starch	+	+	+	+		+	−	−
Skim milk	+	+	w	+		+	NG	−
Gelatin liquefaction	−	+	+	−		+	−	−
**Chemotaxonomic profile**								
Whole-cell sugars	Man, Xyl	Ara	Xyl	Man, Ara, Xyl		Man, Glc	Man, Ara, Glc, Xyl	Xyl
Whole-cell amino acids	d-Glu, Gly, d-Ala, l-Ala, *β*-Ala, l-Orn	d-Glu, Gly, d-Ala, l-Ala, *β*-Ala, l-Orn	d-Glu, Gly, d-Ala, l-Ala, *β*-Ala, l-Orn	d-Glu, Gly, d-Ala, l-Ala, *β*-Ala, l-Orn, l-Ser		d-Glu, Gly, d-Ala, l-Ala, *β*-Ala, l-Orn, l-Ser	d-Glu, Gly, d-Ala, l-Ala, *β*-Ala, l-Orn, l-Ser	d-Glu, Gly, d-Ala, l-Ala, *β*-Ala, l-Orn, l-Ser
DNA G+C content (mol%)	54.3	54.0	51.1	50.3		49.7	49.6	49.7
Phospholipids	PI, PG, DPG, GL, PL	PI, PG, DPG, GL	PI, PG, DPG, GL	PI, PG, DPG, GL, PL, L		PI, PG, DPG, GL, PL, L	PI, PG, DPG, GL, PL, L	PI, PG, DPG, GL, PL, L
Major fatty acids (>10%)	ai-C_17:0_ i-C_17:0_ C_16:1_ 2-OH i-C_16:0_	C_16:1_ 2-OH ai-C_17:0_ i-C_17:0_	i-C_17:0_ C_16:1_ 2-OH	C_16:1_ 2-OH ai-C_17:0_ i-C_17:0_ i-C_16:0_	C_16:1_ 2-OH Sum in feature 9 i-C_16:0_		C_16:1_ 2-OH i-C_17:0_	C_16:1_ 2-OH i-C_17:0_ Sum in feature 9 ai-C_17:0_
Major menaquinone	MK-9(H_2_)	MK-9(H_2_)	MK-9(H_2_)	MK-9(H_2_)	MK-9(H_2_)		MK-9(H_2_)	MK-9

−, absent; +, Present; ai, *anteiso*-branched; Ala, alanine; Ara, arabinose; DPG, diphosphatidylglycerol; G+C, guanin+cytosine; GL, unidentified glycolipid; Glc, glucose; Glu, glutamic acid; Gly, glycine; i, *iso*-branched; I, inhibited; L, unidentified lipid; Man, mannose; NG, no growth; Orn, ornithine; PG, phosphatidylglycerol; PI, phosphatidylinositol; PL, unidentified phospholipids; Ser, serine; w, weak; Xyl, xylose; β-Ala, β-alanine.

## Chemotaxonomic analysis

The freeze-dried cells of the strain S3.2.2.5^T^ were collected for chemotaxonomic analyses after the strain was cultivated in NBRC medium 231 broth for 7–14 days at 30 °C and 100 r.p.m. The polar lipid was extracted according to Minnikin *et al.* [39] and analysed by two-dimensional TLC according to Minnikin *et al.* [40]. The strain was treated in advance using the sonication method to disrupt the cells [41]. The cell-wall amino acid composition was analysed by two-dimensional TLC according to Harper and Davis [42]. The cell-wall sugar composition was analysed using cellulose TLC based on the method described by Hasegawa *et al.* [43]. Major menaquinone was isolated and purified from 100 mg of freeze-dried cells using a chloroform–methanol solvent mixture (2 : 1, v/v) according to the method described by Collins *et al.* [44]. The crude menaquinone extract was purified by TLC 20×20 cm, silica gel 60 F254 (Merck) using petroleum ether-diethyl ether (9 : 1, v/v). The purified quinones were then analysed by reverse-phase HPLC with a mobile phase of methanol and isopropyl ether (3 : 1, v/v). Quinone detection was achieved using a UV detector set at 270 nm. The cellular fatty acid composition was evaluated for the strain S3.2.2.5^T^ and *D. aurantiacus* S-27^T^. The strains were grown in NBRC 231 broth medium at 28 °C with shaking at 100 r.p.m. for 7 days. The fatty acid composition was prepared and analysed based on the instructions of the MIDI Sherlock Microbial Identification System (version 6.5; MIDI) [45] and TSBA6 database.

The chemotaxonomic characters that differentiated strain S3.2.2.5^T^ from the other species within the genus *Dictyobacter* are summarized in Table 2. The main components of whole-cell sugars were xylose and mannose. The major polar lipids were composed of phosphatidylinositol, phosphatidylglycerol, diphosphatidylglycerol, two unidentified glycolipids, and an unidentified phospholipid (Fig. S1). The whole-cell amino acids were d-glutamic acid, glycine, d-alanine, l-alanine, *β*-alanine and l-ornithine. The l-serine was absent. The major cellular fatty acids (>10%) were anteiso-C_17:0_ (24.78%), iso-C_17:0_ (18.24%), C_16:1_ 2-OH (17.42%) and iso-C_16:1_ (14.22%). The major menaquinone of strain S3.2.2.5^T^ was identified as MK-9(H_2_), comprising 81.21% of the total quinone content. The minor quinone was MK-9 (18.78%). The major chemotaxonomic characteristics that differentiate the strain S3.2.2.5^T^ from its closest species *D. aurantiacus* S-27^T^ were major whole-cell sugars and fatty acid profiles. The major whole-cell sugar composition of *D. aurantiacus* S-27^T^ was arabinose, and the major fatty acids were C_16:1_ 2-OH (27.92%), anteiso-C_17:0_ (24.78%) and iso-C_17:0_ (20.4%). Both strains were lack of serine in their whole-cell amino acid. The detailed cellular fatty acid profiles of strain S3.2.2.5^T^ and *D. aurantiacus* S-27^T^ are shown in Table 3.

**Table 3.** Major cellular fatty acids (%) in strains S3.2.2.5^T^ and reference species Strains: 1, S3.2.2.5^T^; 2, *D. aurantiacus* S-27^T^. All data in this table are determined in this study. Cells used in this analysis were grown in NBRC 231 broth medium at 28 °C with shaking at 100 r.p.m. for 14 days. nd, Not detected. Only fatty acids with content greater than 0.5% are shown. Summed feature 3, C_16 : 1_ w7c and/or C_16 : 1_ w6c; Summed feature 8, C_18 : 1_ w7c and/or w6c; Summed feature 9, C_17 : 1_ iso w9c and/or C_16 : 0_ 10-methyl.

Fatty acids (%)	1	2
**Straight chain**		
C_16:0_	3.80	3.29
C_18:0_	2.46	2.41
**Unsaturated**		
C_17:1_ w8c	0.84	0.30
C_18:1_ w9c	1.84	1.58
**Branched**		
C_15:0_ anteiso	0.79	0.56
C_16:1_ iso G	1.36	0.33
C_16:0_ iso	14.22	7.82
C_17:1_ anteiso A	2.69	1.04
C_17:0_ iso	18.24	21.65
C_17:0_ anteiso	24.78	23.75
C_17:0_ 10-methyl	1.50	0.42
C_18:0_ iso	1.41	1.43
C_19:0_ anteiso	1.27	1.80
**Hydroxy**		
C_16:1_ 2-OH	17.42	27.92
**Summed features**		
Summed feature 3	0.64	0.42
Summed feature 8	0.81	0.52
Summed feature 9	4.55	3.20

Based on the results of phenotypic, chemotaxonomic and genomic profiles, strain S3.2.2.5^T^ represents a new member of a new species in the genus *Dictyobacter*, for which the name *D. halimunensis* sp. nov. is proposed.

## Description of *Dictyobacter halimunensis* sp. nov.

*Dictyobacter halimunensis* (ha.li.mun.en’sis. N.L. masc. adj. *halimunensis*, refers to Halimun Mountain, West Java, Indonesia, where the type strain was isolated).

Cells are Gram-stain-positive, aerobic, catalase-positive, non-motile, filamentous and spore-forming and form putative sporangia on aerial mycelia. Growth occurs at 15–37 °C (optimum at 30 °C), at pH 5.0–7.0 (optimum at pH 7.0) and with 0–1% NaCl on NBRC 231 medium. Hydrolyse CMC, MCC, xylan, starch and skim milk. The cell-wall sugars are xylose and mannose. The polar lipids are phosphatidylinositol, phosphatidylglycerol, diphosphatidylglycerol, unidentified glycolipids and unidentified phospholipids. The major menaquinone is MK-9(H_2_). The cell-wall amino acids are d-glutamic acid, glycine, d-alanine, l-alanine, *β*-alanine and l-ornithine. The major fatty acids are anteiso-C_17:0_, iso-C_17:0_, C_16:1_ 2-OH and iso-C_16:1_. The genome size and G+C content are 9.41 Mbp and 54.3 mol%, respectively.

The type strain, S3.2.2.5^T^ (=UICC B-128^T^ = CGMCC 1.61913^T^ = KCTC 43728^T^), was isolated from soil inside a decayed bamboo stem in the forest near the Cisolok geysers, West Java, Indonesia.

The draft whole-genome sequences of the type strain S3.2.2.5^T^ have been deposited in DDBJ under the accession numbers BSRI01000001-BSRI01000003, and the GenBank accession number for the 16S rRNA gene sequence of the type strain S3.2.2.5^T^ and other two strains are LC715230-LC715232, respectively.

## Supplementary material

10.1099/ijsem.0.006600 Uncited Supplementary Material 1.

## Abbreviations

AAs: auxiliary activities
bac120: 120 bacterial single-copy marker genes
CAZymes: carbohydrate-active enzymes
CBMs: carbohydrate-binding modules
CEs: carbohydrate esterases
CMC: carboxymethyl cellulose
dDDH: digital DNA–DNA hybridization
GGDC: Genome-to-Genome Distance Calculator
GHs: glycoside hydrolases
GTs: glycosyl-transferases
ISP: International Streptomyces Project
MCC: microcrystalline cellulose
NaCl: sodium chloride
orthoANI: orthologous average nucleotide identity
R2A: Reasoner’s 2A broth
SEM: scanning electron microscopy
