# Supplementary material for: Dictyobacter halimunensis sp. nov., a new member of the phylum Chloroflexota, from forest soil in a geothermal area
Source: Int J Syst Evol Microbiol. 2024 Dec 4;74(12):006600. doi: 10.1099/ijsem.0.006600 (PMC12453558; doi:10.1099/ijsem.0.006600)
Supplement: Uncited Supplementary Material 1. [file ijsem-74-06600-s001.pdf]

Supplementary Data

Dictyobacter halimunensis sp. nov., a new member of the phylum *Chloroflexota*, from forest soil in a geothermal area

Mazytha Kinanti Rachmania[1,2], Fitria Ningsih[1,2], Dhian Chitra Ayu Fitria Sari[2], Yasuteru Sakai[3,4], Akira Yokota[3,4†], Shuhei Yabe[3,4,5*], Song-Gun Kim[6], and Wellyzar Sjamsuridzal[1,2*]

[1]Department of Biology, Faculty of Mathematics and Natural Sciences, Universitas Indonesia, Kampus UI Depok, 16424, Indonesia

[2]Center of Excellence for Indigenous Biological Resources-Genome Studies, Faculty of Mathematics and Natural Sciences, Universitas Indonesia, Kampus UI Depok, 16424, Indonesia

[3]Department of Microbial Resources, Graduate School of Agricultural Science, Faculty of Agriculture, Tohoku University, 468-1 Aramaki Aza Aoba, Aoba-ku, Sendai, Miyagi, 980-8572, Japan

[4]Hazaka Plant Research Center, Kennan Eisei Kogyo Co.Ltd., 44 Inariyama, Ashitate, Shibata-gun, Miyagi, 989-1311, Japan

[5]BioResource Research Center, RIKEN, 3-1-1 Koyadai, Tsukuba, Ibaraki, 305-0074, Japan

[6]Biological Resource Center / Korean Collection for Type Cultures (KCTC), Korea Research Institute of Bioscience and Biotechnology, Jeongeup, Jeonbuk 56212, Republic of Korea

*** Corresponding authors:**
1. Wellyzar Sjamsuridzal, sjwelly@sci.ui.ac.id
2. Shuhei Yabe, shuhei.yabe@riken.jp

Supplementary data contain one figure and five tables.

Legend to the figures and tables in the supplementary material

Fig S1.

The polar lipid profile of strain S3.2.2.5^{T}. Chromatograms were developed in the first dimension with chloroform/ methanol/water (65:25:4 vol/vol), and the second dimension with chloroform/acetic acid/methanol/water (80:15:12:4 vol/vol). A, Phospomolybdate spray (detection of all lipids); B, Ninhydrin spray (detection of free amino groups; aminolipids); C, Dittmer-Lester reagent (detection of phospolipids); D, p-Anisaldehyde reagent (detection of sugar-containing lipids; glycolipid); E, Dragendorf reagent (detection of phosphatidylcholine); F, Periodate-Schiff (detection of glycol lipid). Abbreviations: PI, phosphatidylinositol; PG, phosphatidylglycerol; DPG, diphosphatidylglycerol; GL, unidentified glycolipid; PL, unidentified phospholipids.

Table S1.

Substrate mycelia colors of strain S3.2.2.5^{T}, S3.2.1.5, and S3.2.1.6 in the various media after being incubated at 30 °C for 21 days. Note: (+), poor; (++), moderate; (+++), abundant; (-), negative.

Table S2.

Pairwise similarity values (%) of the 16S rRNA gene sequences among three *Dictyobacter* strains (S3.2.1.5, S3.2.1.6, and S3.2.2.5^{T}) from Cisolok geothermal area and species members of the genus *Dictyobacter*.

Table S3.

Genome features of *Dictyobacter halimunensis* S3.2.2.5^{T} and other species within the genus *Dictyobacter*. Annotation was completed using the DFAST pipeline (version1.6.0). Genome completeness and contamination levels were estimated with CheckM in DFAST.

Table S4.

The secondary metabolic gene clusters of the strain S3.2.2.5^{T} predicted by antiSMASH.

Table S5.

Composition of CAZymes annotated from the genome of *Dictyobacter halimunensis* S3.2.2.5^{T} and *D. aurantiacus* S-27^{T}. Data were obtained from the dbCAN2 meta server. Abbreviations: GH, glycoside hydrolase; CBM, carbohydrate-binding modules; CE, carbohydrate esterases; AA, auxiliary activities; GT, glycosyl-transferases; PL, polysaccharide lyases.

Supplementary Figure S1.

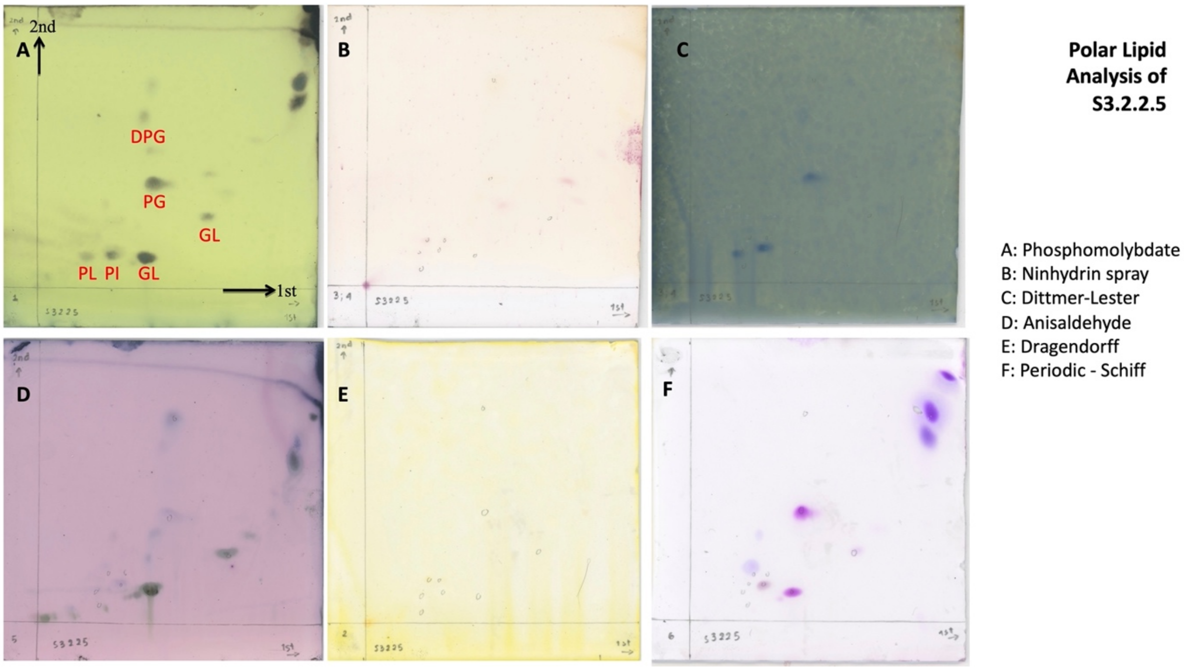

Fig S1.

The polar lipid profile of strain S3.2.2.5^{T}. Chromatograms were developed in the first dimension with chloroform/ methanol/water (65:25:4 vol/vol), and the second dimension with chloroform/acetic acid/methanol/water (80:15:12:4 vol/vol). A, Phospomolybdate spray (detection of all lipids); B, Ninhydrin spray (detection of free amino groups; aminolipids); C, Dittmer-Lester reagent (detection of phospolipids); D, p-Anisaldehyde reagent (detection of sugar-containing lipids; glycolipid); E, Dragendorf reagent (detection of phosphatidylcholine); F, Periodate-Schiff (detection of glycol lipid).

Abbreviations: PI, phosphatidylinositol; PG, phosphatidylglycerol; DPG, diphosphatidylglycerol; GL, unidentified glycolipid; PL, unidentified phospholipids.

Supplementary Table S1.

Table S1. Substrate mycelia colors of strain S3.2.2.5^{T}, S3.2.1.5, and S3.2.1.6 in the various media after being incubated at 30 °C for 21 days. Note: (+), poor; (++), moderate; (+++), abundant; (-), negative.

**Isolate code**	**S3.2.2.5^{T}**	**S3.2.1.5**	**S3.2.1.6**
ISP 1 + 2 % gellan gum	++	++	++
Colony colour	Yellow orange	Orange	Orange
1/10 ISP 1 + 2 % gellan gum	++	++	++
Colony colour	Orange	Orange	Orange
ISP 3 + 2 % gellan gum	+	+	+
Colony colour	Orange	Orange	Orange
1/10 ISP 3 + 2 % gellan gum	+	+	+
Colony colour	Orange	Yellow orange	Yellow orange
R2A + 2 % gellan gum	-	-	-
Colony colour	No growth	No growth	No growth
1/10 R2A + + 2 % gellan gum	+	+	+
Colony colour	Pale orange	Yellow orange	Yellow orange
FS1V + 2 % gellan gum	+	+	+
Colony colour	Light yellow orange	Yellow orange	Orange
1/10 FS1V + + 2 % gellan gum	+	+	+
Colony colour	Light yellow orange	Yellow orange	Orange
NBRC 231 + 2 % gellan gum	+++	+++	+++
Colony colour	Orange	Orange	Orange
1/10 NBRC 231 + 2 % gellan gum	+	+	+
Colony colour	Yellow orange	Yellow orange	Orange

Supplementary Table S2.

Table S2. Pairwise similarity values (%) of the 16S rRNA gene sequences among three *Dictyobacter* strains (S3.2.1.5, S3.2.1.6, and S3.2.2.5^{T}) from Cisolok geothermal area and species members of the genus *Dictyobacter*.

**Strain code**	**S3.2.1.5**	**S3.2.1.6**	**S3.2.2.5^{T}**
S3.2.1.5	100.00		
S3.2.1.6	99.85	100.00	
S3.2.2.5^{T}	99.92	99.92	100.00
D. aurantiacus S-27 (LC210808)	98.34	98.41	98.34
D. kobayashii Uno11 (LC278466)	96.90	96.90	96.90
D. alpinus Uno16 (LC278467)	96.30	96.37	96.30
D. vulcani W12 (LC422201)	96.22	96.30	96.22
D. arantiisoli Uno17 (LC278468)	96.30	96.37	96.30
D. formicarum SOSP1-9 (AM180155)	96.37	96.37	96.37
T. tsumagoiensis Uno3 (LC278465)	91.69	91.69	91.69

Supplementary Table S3.

Table S3. Genome features of *Dictyobacter halimunensis* S3.2.2.5^{T} and other species within the genus *Dictyobacter*. Annotation was completed using the DFAST pipeline (version 1.6.0). Genome completeness and contamination levels were estimated with CheckM in DFAST.

**Species**	**GenBank Assembly Accession**	**Genome size (Mbp)**	**G+C content (mol%)**	**Total contigs**	**Completeness (%)**	**Contamination (%)**	**Total CDS**	**Number of rRNA**	**Number of tRNA**	**Number of CRISPRs**
D. halimunensis S3.2.2.5^{T}	GCA_036245075	9.41	54.3	3	98.28	6.58	8030	27	65	3
D. formicarum SOSP1-9^{T}	GCA_016587435	9.21	51.1	63	96.39	4.39	7958	25	59	5
D. alpinus Uno16^{T}	GCA_003967575	8.96	49.7	4	100.0	2.66	7719	28	64	6
D. aurantiacus S-27^{T}	GCA_003967515	8.88	54.0	2	100.0	3.29	7470	27	67	8
D. kobayashii Uno11^{T}	GCA_003967555	8.85	50.3	2	100.0	2.82	8316	27	64	4
D. vulcani W12^{T}	GCA_008974265	7.42	49.7	7	98.12	2.66	6448	28	64	8
D. arantiisoli Uno17^{T}	GCA_008326305	7.21	49.7	256	98.28	0.00	5628	11	67	11

Supplementary Table S4.

Table S4. The secondary metabolic gene clusters of the strain S3.2.2.5^T predicted by antiSMASH.

**Contig**	**Region**	**Position**	**Types of secondary metabolite clusters**	**Most similar known cluster**	**MIBiG accession**	**Similarity**	**Reference**
	1.1	462,813-508,604 nt. (total: 45,792 nt)	T1PKS	No matches found			
	1.2	865,937-988,063 nt. (total: 122,127 nt)	T1PKS, NRPS, Prodigiosin	puwainaphycin F / minutissamide A / minutissamide B / minutissamide C / minutissamide D from *Anabaena* sp. UHCC-0399	BGC0001953	23%	(1)
1	1.3	1,038,003-1,061,048 nt. (total: 23,046 nt)	Lanthipeptide-class-II, Proteusin	No matches found			
	1.4	1,609,238 - 1,629,504 nt. (total: 20,267 nt)	Lanthipeptide-class-II	No matches found			
	1.5	2,126,918 - 2,150,217 nt. (total: 23,300 nt)	Lanthipeptide-class-II	No matches found			
	1.6	2,786,720 - 2,867,907 nt. (total: 81,188 nt)	LAP,NRPS	Nostocyclopeptide A2 from *Nostoc* sp. ATCC 53789	BGC0000397	28%	(2)
	1.7	2,941,265 - 2,970,459 nt. (total: 29,195 nt)	LAP	No matches found			
	1.8	3,089,058 - 3,100,944 nt. (total: 11,887 nt)	RiPP-like	No matches found			
	1.9	3,130,214 - 3,140,465 nt. (total: 10,252 nt)	Melanin	No matches found			
	2.1	3,239,724 - 3,260,686 nt. (total: 20,963 nt)	Terpene	No matches found			
2	2.2	3,610,469 - 3,665,044 nt. (total: 54,576 nt)	NRPS,NRPS-like,T1PKS	rakicidin A / rakicidin B from *Micromonospora purpureochromogenes*	BGC0001327	22%	(3)
	2.3	4,746,787 - 4,771,229 nt. (total: 24,443 nt)	Lanthipeptide-class-II, Proteusin	No matches found			
	2.4	5,040,947 - 5,068,303 nt. (total: 27,357 nt)	Lanthipeptide-class-II	No matches found			

Supplementary Table S5.

Table S5. Composition of CAZymes annotated from the genome of *Dictyobacter halimunensis* S3.2.2.5^{T} and *D. aurantiacus* S-27^{T}. Data were obtained from the dbCAN2 meta server. Abbreviations: GH, glycoside hydrolase; CBM, carbohydrate-binding modules; CE, carbohydrate esterases; AA, auxiliary activities; GT, glycosyl-transferases; PL, polysaccharide lyases.

Strain	Genome size (Mb)	Proteins	CAZymes	GH	CBM	CE	AA	GT	PL
D. halimunensis S3.2.2.5^{T}	9.41	8030	307	138	19	36	15	96	3
D. aurantiacus S-27^{T}	8.88	7470	266	110	22	28	16	90	0

References

1. Mareš J, Hájek J, Urajová P, Kust A, Jokela J, Saurav K, et al. Alternative Biosynthetic Starter Units Enhance the Structural Diversity of Cyanobacterial Lipopeptides. Appl Environ Microbiol. 2019;85(4):e02675-18.
2. Luesch H, Hoffmann D, Hevel JM, Becker JE, Golakoti T, Moore RE. Biosynthesis of 4-Methylproline in Cyanobacteria: Cloning of nosE and nosF Genes and Biochemical Characterization of the Encoded Dehydrogenase and Reductase Activities. J Org Chem. 2003;68(1):83–91.
3. Tsakos M, Jakobsen KM, Yu W, Villadsen NL, Poulsen TB. The Rakicidin Family of Anticancer Natural Products – Synthetic Strategies towards a New Class of Hypoxia-Selective Cytotoxins. Synlett. 2016;27(13):1898–906.
